# Pulmonary metastatectomy in pediatric cancer patients at National Cancer Institute, Egypt: prognostic factors and outcome

**DOI:** 10.1186/s43046-023-00198-9

**Published:** 2024-01-22

**Authors:** Asmaa Hamoda, Inas Elattar, Heba Mahmoud, Mohamed Abdelrahman, Emad Ebied

**Affiliations:** 1 Department of Pediatric Oncology, National Cancer Institute/ Cairo University, Cairo, Egypt; 2 Department of Pediatric Oncology, Children Cancer Hospital Egypt, Cairo, Egypt; 3 Hemato-Oncology Department at Children Cancer Hospital of Egypt (57357), Cairo, Egypt; 4 Department of Statistics, National Cancer Institute, Cairo University, Cairo, Egypt; 5 Department of Pediatric Oncology, Naser Institute, Cairo, Egypt; 6 Department oF Surgical Oncology, National Cancer Institute, Cairo University, Cairo, Egypt

**Keywords:** Pediatric solid tumours, Wilms tumour, Osteosarcoma, Lung metastases, Pulmonary nodules, Pulmonary metastatectomy

## Abstract

**Background:**

Metastatic tumors account for 80% of all lung tumors in children. Wilms tumour and osteosarcoma are the most tumors of childhood that produce lung metastases. The aim of the current study is to assess the prognostic factors of pulmonary metastatectomy in pediatric solid tumours as age, number, size, site,laterality, resectability of pulmonary nodules, and number of Thoracotomies. Calculate overall survival among patients who underwent pulmonary metastatectomy.

**Methods:**

It is a retrospective study including all pediatric patients with metastatic solid tumors to lungs treated at pediatric oncology department, National Cancer Institute, Cairo University from 2008 to 2014. Fifty-five patients were included, 43 (78.2℅) patients of them had Osteosarcoma.

**Results:**

Thirty (54.5℅)patients were male. The mean age was 15 years ranging from (4.5- 23) years. The site of primary disease was at lower limbs in 43 (78.2%) patients. All patients underwent complete surgical resection of the primary disease with negative margin, 22(51.1%) of the osteosarcoma patients did amputation with tumor necrosis less than 90%. All patients received chemotherapy and only 9 received radiation therapy. The patients were classified into four groups according to time of diagnosis of pulmonary metastasis: at time of diagnosis in 13 (21.8%) patients, within treatment in 16 (30.9%) patients, within first year follow up in 18 (32.7%) patients and detected late in 8 (14.5%) patients. Bilateral lung metastasis diagnosed by CT chest were detected in 42 (76.4%) patients. Size of metastatic nodules was ranging from (0.5 to 10 cm) with mean 3.4 cm. Number of metastatic nodules was ranging from (1 to 28) median 4.Metastatic complications were detected in 19 patients. 5-year OS was 74.8% in the study group, and 68% in osteosarcoma patients. Effect of prognostic factors as sex, time of respectability, laterality, tumor necrosis of the 1ry disease, Timing of lung metastasis, size and site of the primary, Surgical approach of metastatectomy, postoperative complications on overall survival of the studied patients was done with significant *P*-value of tumor necrosis of the 1ry disease and Timing of lung metastasis 0.017, 0.001 respectively.

**Conclusion:**

Resection of pulmonary metastases of pediatric solid tumours is a safe and effective treatment that offers better survival.

## Introduction

Lung is one of the most common organ where metastatic disease is found for many malignancies. Some lesions are discovered due to symptoms such as pneumonia, cough, hemoptysis or pain, but most are asymptomatic and are found on routine staging or surveillance imaging [1]. As metastasis is a disseminated process, treatment depends on effective systemic therapy, but surgical resection can sometimes be therapeutic. Several reports have shown prolonged survival after pulmonary metastasectomy in selected patients presenting with isolated lung metastases, mainly in case of osteosarcoma; however prognostic factors are still unclear [2].

Tronc et al*.* reported that pulmonary metastasectomy is a safe and potentially curative treatment in pediatric patients presenting with secondary lung lesions form solid tumours of different histology [3]. In some cases, the surgery is therapeutic, and in some it plays a diagnostic role and guides further systemic treatment. In general, the more resistant a histology to systemic treatment the more central a role metastasectomy plays in cure [4].

A more recent study done by Memorial Sloan Kettering Cancer Center, USA, about pulmonary metastasectomy in pediatric solid tumors they concluded that, although management of metastatic disease relies heavily on systemic therapies, surgery plays an important role. In some cases, the surgery is therapeutic, and in some it plays a diagnostic role and guides further systemic treatment. In general, the more resistant a particular histology is to systemic treatment the more central a role metastasectomy plays in cure [4].

## Aim of work

This study aim is the assessment of prognostic factors of pulmonary metastatectomy in pediatric solid tumors and calculate overall survival (OS) among patients who underwent pulmonary metastatectomy. Correlation of different prognostic factors with overall survival.

## Patients and methods

This is a retrospective study that included patients at Pediatric Oncology Department, National Cancer Institute (NCI), Cairo University during the period from January 2008 to December 2014.

The current study included fifty-five patients who underwent pulmonary metastatectomy during this period who were diagnosed to have isolated lung metastasis from different solid tumors with completely excised primary disease. The study included all pediatric patient with solid tumors metastatic to lungs, with resectable primary tumor, with good performance status, and in addition, resectability of lung disease was assessed based on C.T chest (computed tomography of chest).

All patients had their medical records reviewed for history and Clinical examination; this should include documentation of age, sex, date of primary disease diagnosis, date of appearance of lung metastasis, date of last follow up, symptoms, measurement of primary tumour size and data from pathology report regarding the excised lung nodules as number, surgical margin, size, and histological diagnosis. All patients underwent complete blood count (CBC), Liver and Kidney functions tests (LFT &KFT), alkaline phosphatase (ALP), lactate dehydrogenase (LDH), creatinine clearance, alfa-feto protein, serum tumour marker of germ cell tumour. Radiological imaging as PA, lateral and two oblique X-RAY especially for bone tumours, CT chest without contrast to detect metastasis was performed initially and repeated during therapy at different checkpoints according to the protocol implemented, CT-chest 3 mm cuts, which is the most widely used investigation to diagnose pulmonary metastasis, was done for all patients. Radiological characteristics were analysed in each patient as follows: the number of pulmonary nodules, the size of the largest nodule, the laterality of the nodules and pleural involvement. Computed tomography of abdomen and pelvis with contrast for proper staging is needed. Magnetic Resonant Imaging for primary tumours especially bone tumours and other suspicious sites, was performed initially and was repeated during therapy and at certain checkpoints according to the protocol implemented. 99technetium bone scan was done for detection of bone metastasis. Echocardiogram before Doxorubicin containing cycles was done.

True cut biopsy (for the initial disease) guided by CT was usually sufficient otherwise open biopsy if the needle biopsy was not conclusive or inadequate. Retrieval of all paraffin blocks for the primary tumours and for surgically excised lung nodules was done. Detection of the percentage of therapy response of primary tumours as well as state of resection margins of the primary disease and metastatic lung nodules post chemotherapy was done.

There were two procedures for pulmonary metastasectomy, thoracotomy or video-assisted thoracoscopy (VAT).

### Histopathology and imunohistochemistry

Retrieval of all paraffin blocks for the primary tumours (excess osteoid in osteosarcoma, round cell tumours in Ewing sarcoma and RMS, mesenchymal spindle cells in synovial sarcoma and nephrogenic blastemal, epithelial cells in Wilm ‘s tumor) and for surgically excised lung nodules was done. Detection of the percentage of therapy response of primary tumours as well as state of resection margins of the primary disease and metastatic lung nodules. Diagnostic markers as CD 99 in Ewing sarcoma, myogenin and desmin in Rhabdomyosarcoma, cytokeratin in synovial sarcoma.

Three pathological terms were used to describe the surgical margins of the resected metastatic nodules, Negative margin which means that no cancer cells detected microscopically at the outer edge of the tissue that was removed (greater than 5 mm clearance from the tumour). Positive margin means that cancer cells or tumour extends to the edge of the sample. Close margin means that cancer cells are close to the edge of the tissue that was removed but not right at the edge.

Multidisciplinary treatment involving paediatric oncologists, surgeons, and radiation oncologists is necessary to obtain positive results in children who have pulmonary metastases of oncological diseases, and this differs from disease to another.

For patients with pulmonary metastases, a multimodality approach that includes chemotherapy and supplemental low-dose whole lung irradiation, surgical resection reserved for lung metastases that didn’t resolve with chemotherapy [5].

## Statistical analysis

Data were coded and entered using the statistical package SPSS (Statistical Package for the Social Sciences) version 25. Data was summarized using mean, standard deviation, median, minimum, and maximum in quantitative data and using frequency (count) and relative frequency (percentage) for categorical data. Comparisons between quantitative variables were done using the non-parametric Kruskal–Wallis and Mann–Whitney tests [6]. For comparing categorical data, Chi square test was performed. Exact test was used instead when the expected frequency is less than 5 [7]. Correlations between quantitative variables were done using Spearman correlation coefficient [8]. Survival curves were plotted by the Kaplan–Meier method [9]. *P*-values less than 0.05 were considered as statistically significant.

*Overall survival (OS)*: Overall survival was calculated from the date of diagnosis till the date of death or date of last follow up [10].

*Metastatic free period* is the interval between diagnosis of the primary in initially localized tumor and the metastatic diseases [11].

## Results

**Fig. 1:**
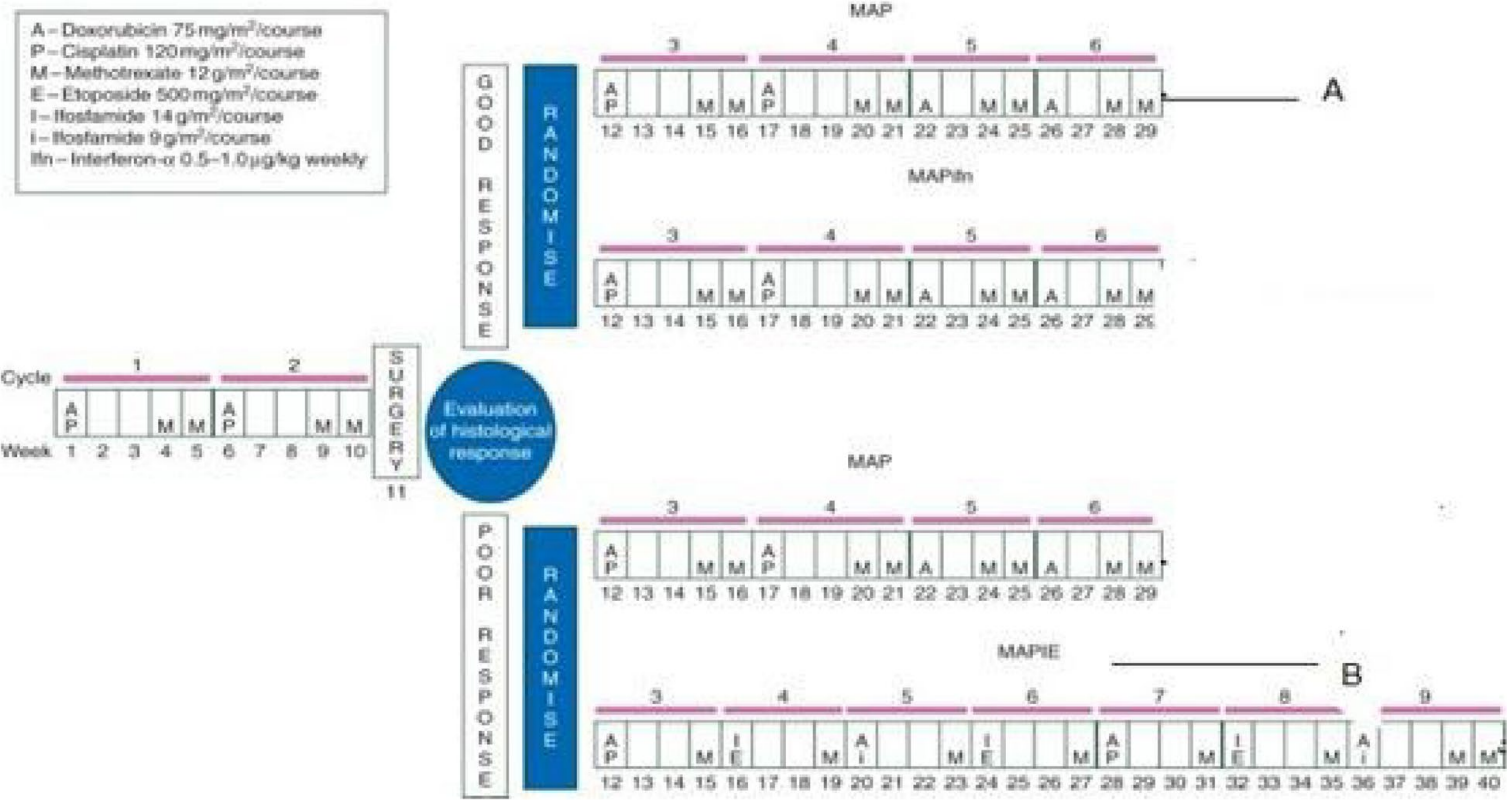
EURAMOS protocol Roadmap

**Fig. 2:**
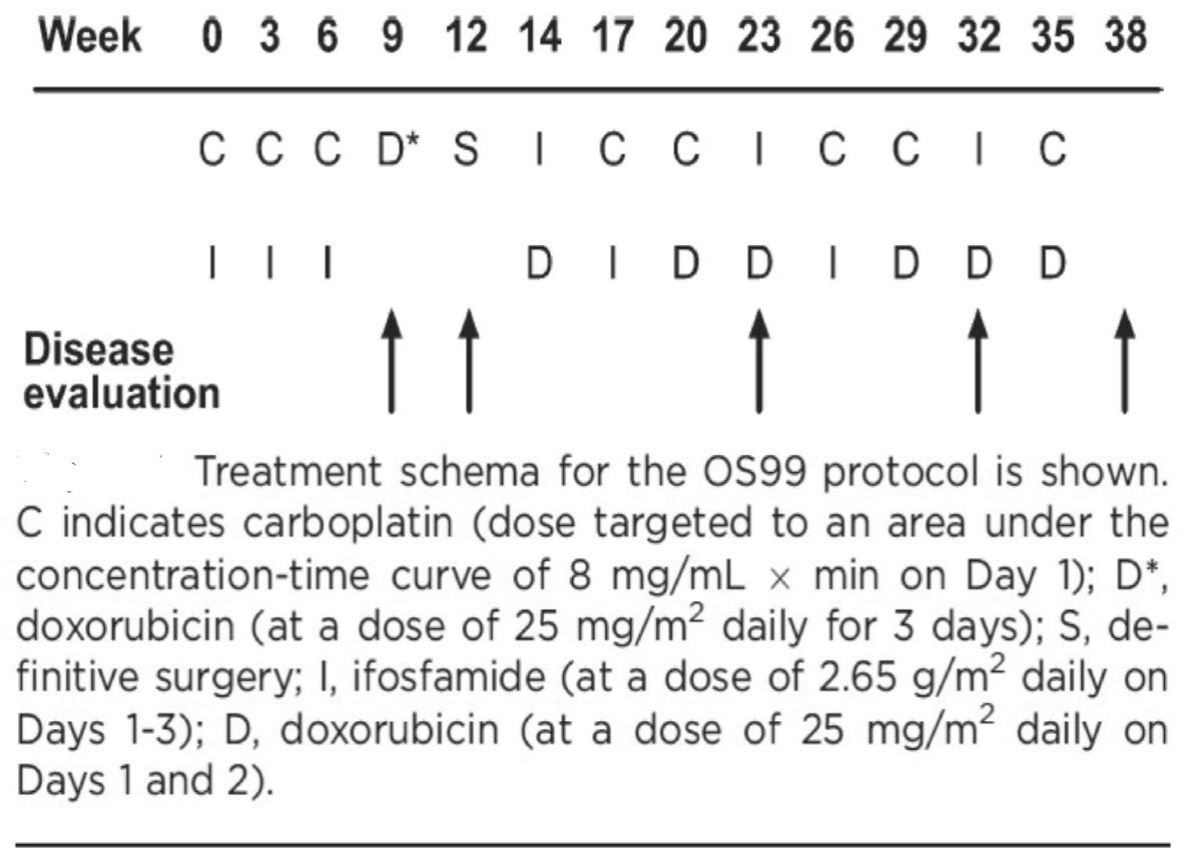
OS 99 protocol Roadmap

**Fig. 3:**
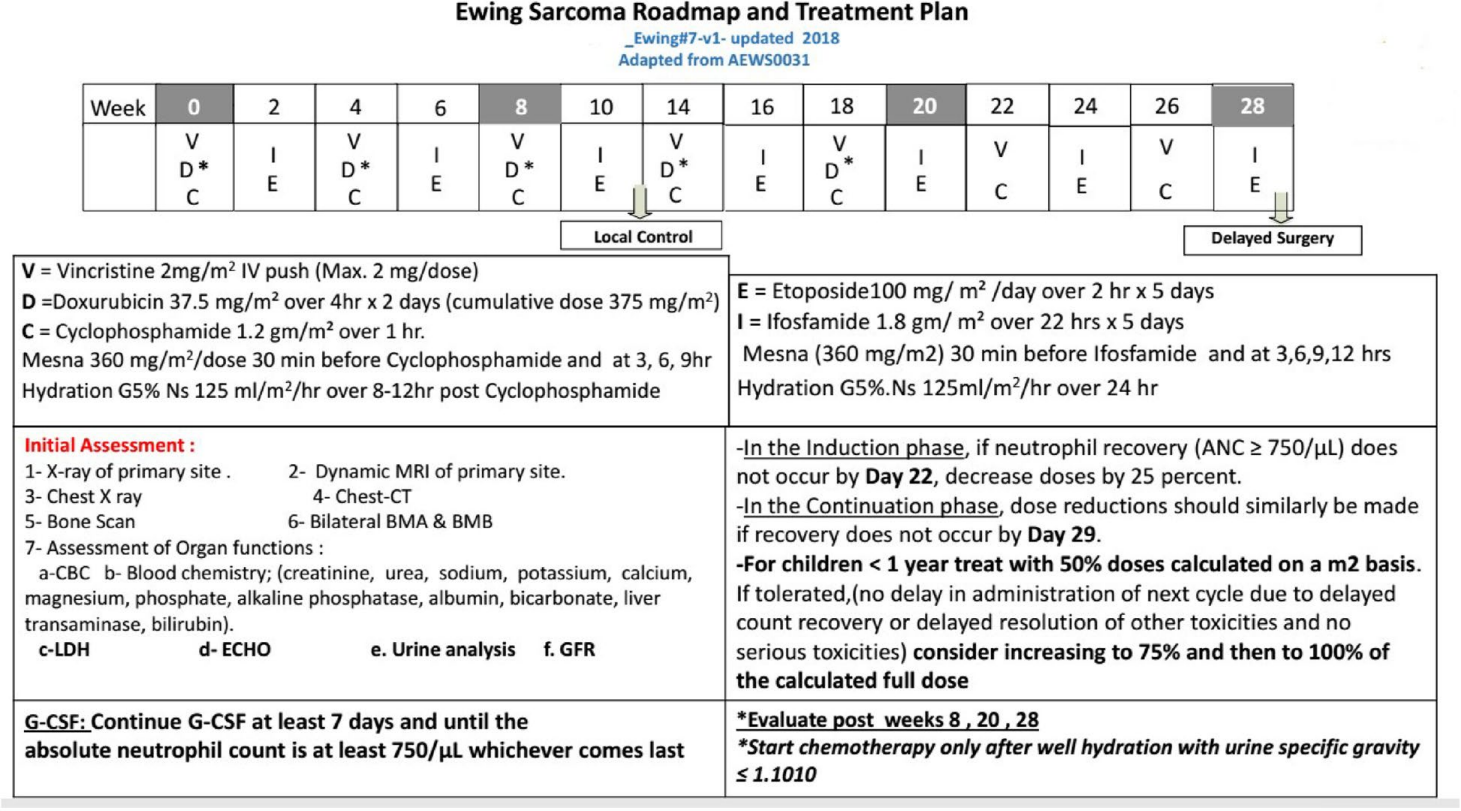
Ewing Sarcoma Roadmap

Fifty-five patients were included who underwent pulmonary metastatectomy during the period between January 2008 to December 2014 at Pediatric Oncology Department, NCI Cairo, 43 (78.2℅) had Osteosarcoma, 5 (9.1℅) cases had Synovial sarcoma, 2 (3.6℅) cases suffering from Rhabdomyosarcoma, 2 (3.6℅) cases diagnosed Ewing Sarcoma,2 (3.6℅) cases had Germ cell tumour, and 1(1.8℅) case had Wilms tumour. There were 30 male (54.5℅) and 25 female 45.5%. The mean age was 15 years ranging from (4.5- 23) years, Table 1. The site of primary disease was at lower limbs in 43 patients (78.2%), upper limbs in 5 patients and other sites as anterior chest wall, axillary area, paraspinal area and kidney, Table 1. All of them underwent complete surgical resection of the primary disease with negative margin in 18 (32.7%) patients, while19 (34.5 %) patients their primary diseases were excised with positive margin. Eighteen (32.7%) patients the primary sites were excised with close margins (this group is considered incomplete resection as positive margin). Twenty-two (51.1%) of the osteosarcoma patients did amputation with tumour necrosis less than 90%, Table 1. All patients received chemotherapy and only 9 received radiation therapy. Out of the 43 Osteosarcoma patients involved in the study, 33 patients received EURAMOS (European and American Osteosarcoma) protocol, Fig. 1, while 4 patients received 0S99 protocol, Fig. 2, and the rest of the patients received cycles of Doxorubicin and cisplatin alternating with cycles of Etopside and Ifosfamide, Fig. 3.

**Table 1:** Primary disease classification and characteristics

		**No**	**%**
**Pathology**	**Osteosarcoma**	43	79.8%
	**Synovial sarcoma**	5	9.1%
	**Ewing sarcoma**	2	3.6%
	**Rhabdomyosarcoma**	2	3.6%
	**Germ cell tumor**	2	3.6%
	**Wilm’s tumor**	1	1.8%
**Site of primary disease**	**Lower limb**	43	78.2%
	**Upper limb**	5	9.1%
	**Axillary mass**	1	1.8%
	**Anterior abdominal wall**	1	1.8%
	**Lung**	1	1.8%
	**Paraspinal**	1	1.8%
	**posterior mediastinum**	1	1.8%
	**Anterior chest wall**	1	1.8%
	**Right kidney**	1	1.8%
**Type of local control**	**Wide local resection**	33	60.0%
	**Amputation**	22	40.0%

**Fig. 4:**
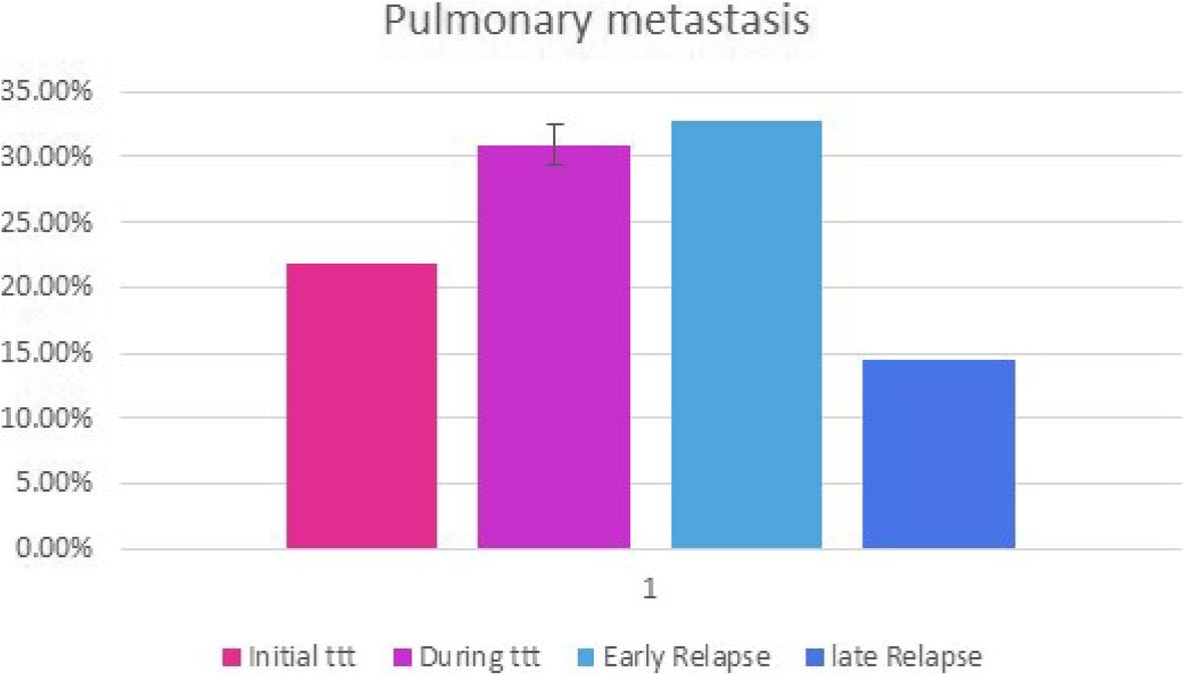
Timing of pulmonary metastasis

The patients were classified into four groups according to time of diagnosis of pulmonary metastasis: at time of diagnosis in 13 (21.8%) patients, within treatment in 16 (30.9%) patients, within first year follow up in 18 (32.7%) patients and detected late in 8 (14.5%) patients. Pulmonary metastases were diagnosed by Ct chest. Unilateral metastasis in 13 (23.6%) patients and bilateral metastasis in 42 (76.4%) patients, Fig. 4.

Thoracotomy was performed in 50 (90.9%) patients, while Video-assisted thoracoscopy (VAT) was performed in 2 (3.6%) patients, and both thoracotomy and VAT were performed in 3 patients (5.5%). some patients did repeated thoracotomies either sequential for bilateral lung metastases or for recurrent lung metastasis. The mean number of thoracotomies was 2, it was ranging from 1 to 6. Size of metastatic nodules was ranging from (0.5 to 10 cm) with mean 3.4cm. The number of metastatic nodules ranged from (1 to 28) median 4. Forty-five (81.8%) patients underwent metastasectomy (wedge resection) which is the resection of the metastatic nodule with surrounding wide surgical margin. Lobectomy was done in 8 (14.5%) patients and only 2 (3.6%) patients underwent pneumonectomy. Almost all patients 54 (98.1%) did metachronous metastasectomy and only one patient (posterior mediastinal Germ cell tumor) did synchronous metastasectomy.

There were no perioperative deaths, complications were detected in 19 patients in the form of restrictive lung disease, pneumothorax, diaphragmatic and haemorrhagic pleural effusion, lung hematoma complications.

**Fig. 5:**
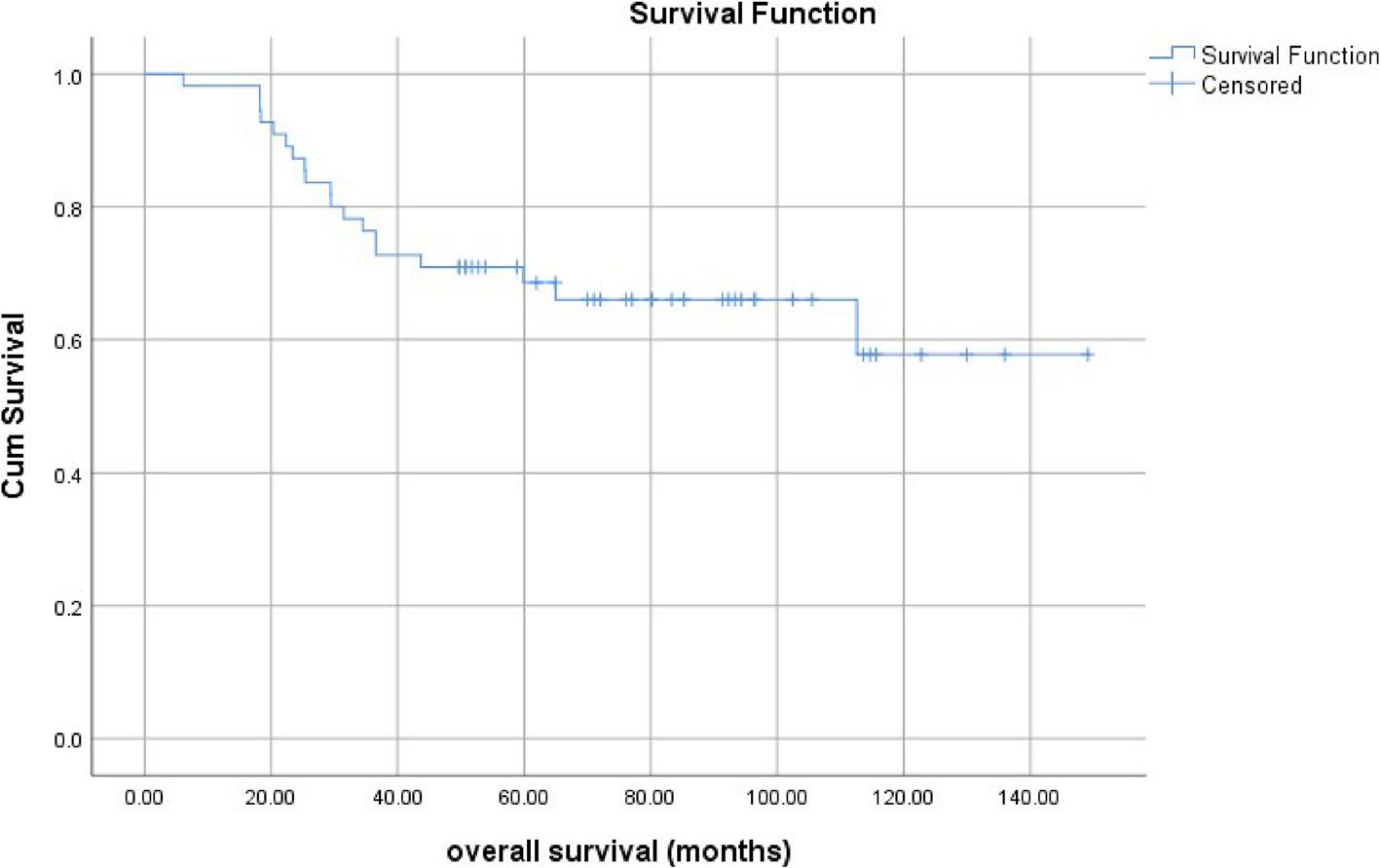
Kaplen-miere for overall survival

**Fig. 6:**
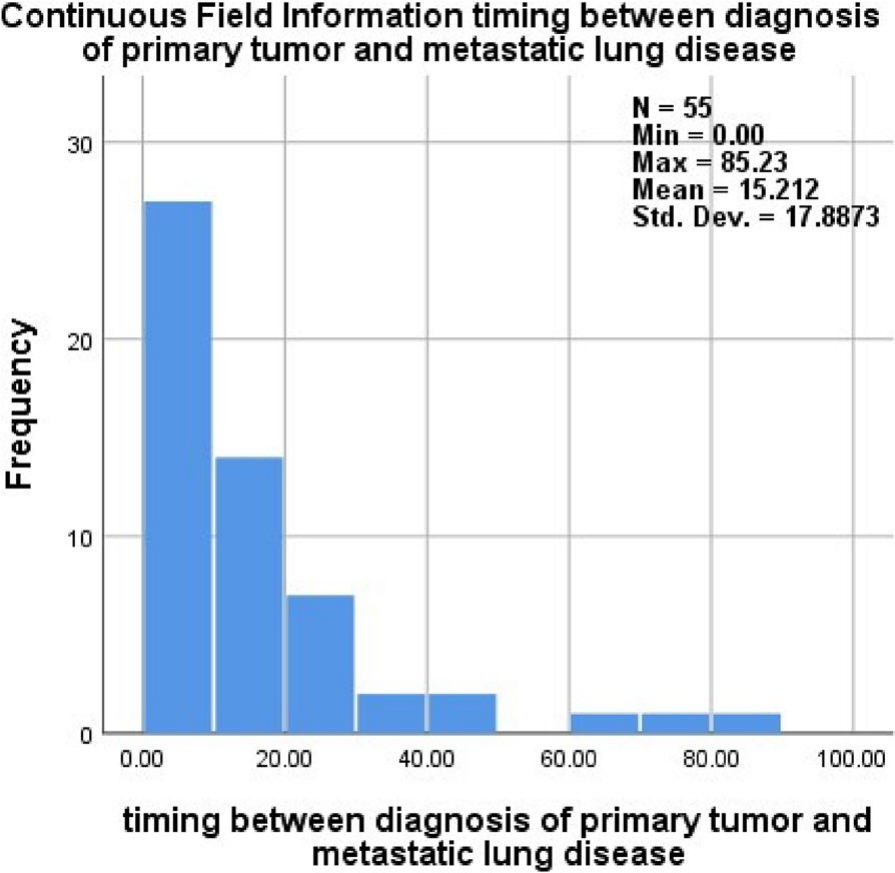
Metastatic free period

Using the date of diagnosis of the primary disease as starting point the OS time was 64 months as median ranging from (6 to 147) months with 5-year overall survival was 74.8%, and the 5-year overall survival in Osteosarcoma patients was 68%, Figs. 5 and 6.

Effect of prognostic factors as sex, time of respectability, laterality, tumor necrosis of the 1ry disease, timing of lung metastasis, size and site of the primary, surgical approach of metastatectomy, postoperative complications on overall survival of the studied patients was done with significant *P*-value of tumour necrosis of the 1ry disease and timing of lung metastasis 0.017, 0.001 respectively, Table 2.

**Table 2:** Effect of prognostic factors on OS of the studied patients

		**Overall survival (months)**					
		**Mean**	**Standard Deviation**	**Median**	**Minimum**	**Maximum**	***P*** ** value**
**Sex**	**Male**	65.48	38.22	60.86	6.10	149.07	0.648
	**Female**	68.82	31.42	69.97	18.20	122.80	
**Time of respectability**	**synchronous**	115.67		115.67	115.67	115.67	0.182
	**metachronous**	66.10	34.69	63.42	6.10	149.07	
**Laterality**	**Unilateral**	81.49	33.38	85.23	36.53	135.97	0.076
	**Bilateral**	62.51	34.67	60.86	6.10	149.07	
**Tumor necrosis of 1ry**	**> or equal 90%**	90.32	31.17	88.78	50.73	129.93	**0.017**
	**< 90%**	58.51	33.32	52.24	6.10	149.07	
**Timing of lung metastasis**	**Initially(synchronous)**	64.59	36.21	61.88	6.10	115.67	**0.001**
	**during treatment**	49.78	30.48	49.73	18.20	102.43	
	**Relapse before 1 year follow up**	64.03	23.47	63.42	31.47	112.57	
	**Relapse after 1 year follow up**	113.89	25.93	118.74	71.03	149.07	
**Size of 1ry**	**< 5 cm**	80.13		80.13	80.13	80.13	0.727
	**> 5 cm**	66.76	35.30	63.42	6.10	149.07	
**Site of 1ry**	**Lower limb**	63.85	34.72	61.90	6.10	149.07	0.318
	**Upper limb**	50.53	27.17	36.53	29.40	96.40	
**Surgical approach of metastatectomy**	**Thoracotomy**	66.19	36.13	63.42	6.10	149.07	0.625
	**Thoracotomy + video-assisted**	81.16	24.60	94.33	52.77	96.37	
	**Video-assisted**	65.91	20.10	65.91	51.70	80.13	
**Complications**	**Yes**	62.90	38.04	59.83	18.23	129.93	0.512
	**No**	69.16	33.67	64.95	6.10	149.07	

N.B: synchronous means surgical resection of both primary disease and pulmonary metastasis at same time

Effects of prognostic factors as sex, age, type of local control, and laterality (limb salvage/amputation) on metastatic free period ( less or more than one year) for the studied patients were done showing significant *P*-value 0.027 as metastatic free period is prolonged to be more than one year with limb salvage than amputation, Table 3.

**Table 3:** Effect of prognostic factors on metastatic free period for the studied patients

		**Metastatic Free Period (grouped)**				***P*** **-value**
		**< = 1 year**		**> 1 year**		
		**N**	**%**	**N**	**%**	
**Sex**	**Male**	18	60.0%	12	40.0%	0.373
	**Female**	12	48.0%	13	52.0%	
**Type of local control**	**Limb salvage**	14	42.4%	19	57.6%	**0.027**
	**Amputation**	16	72.7%	6	27.3%	
**Laterality**	**Unilateral**	5	38.5%	8	61.5%	0.183
	**Bilateral**	25	59.5%	17	40.5%	
		**Metastatic Free Period (grouped)**				***P*** **-value**
		**< = 1 year**		**> 1 year**		
		**Mean**	**Standard Deviation**	**Mean**	**Standard Deviation**	
**Age (years)**		15.4	4.0	14.7	3.8	0.512

Effect of prognostic factors as sex, time of resection, laterality, timing of lung metastasis, tumour necrosis of the primary which is > or equal 90% or < 90%, surgical complications on outcome of the studied patients showing impact of tumour necrosis of the primary, surgical complications on the outcome of the patients with significant *P*-value 0.015, 0.040 respectively, Table 4.

**Table 4:** Effect of prognostic factors on outcome of the studied patients

		**Fate**					***P*** ** value**
		**Alive**		**Dead**			
		**Count**	**%**	**Count**		**%**	
**Sex**	**Male**	17	47.2%	13		68.4%	0.133
	**Female**	19	52.8%	6		31.6%	
**Time of resectability**	**Synchronous**	1	2.8%	0		.0%	1
	**Metachronous**	35	97.2%	19		100.0%	
**Laterality**	**Unilateral**	11	30.6%	2		10.5%	0.180
	**Bilateral**	25	69.4%	17		89.5%	
**Timing of lung metastasis**	**Initially**	9	25.0%	3		15.8%	0.072
	**During treatment**	9	25.0%	8		42.1%	
	**Relapse before 1 year follow up**	10	27.8%	8		42.1%	
	**Relapse after 1 year follow up**	8	22.2%	0		.0%	
**Tumor necrosis of 1ry**	**> or equal 90%**	8	27.6%	0		.0%	**0.015**
	**< 90%**	21	52.4%	19		100.0%	
**Complications**	**Yes**	9	25.0%	10		52.6%	**0.040**
	**No**	27	75.0%		9	47.4%	

NB:

• The statistical method used in Table 2 was according to Kaplan and Meier analysis & Chi square statistics

• Table 3

• Limb salvage is better as it saved the limb which is also psychologically important

## Discussion

Children with solid tumours have 25% metastatic disease at initial diagnosis and another 20% develop metastases during or after treatment. The most common location of these metastases is the lung [4].

Pulmonary metastasectomy is currently indicated for patients with the following criteria: primary tumor controlled, possibility of complete resection verified by computed tomography (CT) of the chest, pulmonary function and performance status compatible with the proposed lung resection, and lack of another available treatment that would be more effective than the surgical procedure [9].

In the current study, most of the patients were diagnosed as Osteosarcoma (79.8℅) followed by Synovial sarcoma (9.1℅), then Rhabdomyosarcoma (3.6℅), Ewing Sarcoma (3.6℅), Germ cell tumors (3.6℅) and Wilm’s tumor (1.8℅). The 5-year overall survival of of the patients in the current study patients was (74.8%). The 5-year overall survival in Osteosarcoma patients was 68%.

The mean age of the studied patients was 15 years old ranging from (4.5- 23) years with males (54.5%) predominance over females (45.5%) There was no statistically significant difference between the age and gender of the patients and overall survival rates (5 year OS) *P* value (0.894) *P* value (0.648) respectively. This agrees with Erginel et al*.* (2016) Turkish study which retrospectively reviewed the medical records of 43 children who were operated on in the Pediatric Surgery Clinic between January 1988 and 2014,forty-three children (26 boys; 17 girls; mean age 10 ± 4.24 years, range (6 months–18 years) who underwent pulmonary metastasectomy, there was statistically significant difference regarding the age while insignificant regarding the gender of the patients and overall survival rates (*p* = 0.029 and *p* = 0.48, respectively) [12].

In the current study, all patients received chemotherapy as per protocol for the primary diseases. Tumour necrosis which represent response of primary disease to chemotherapy was less than 90% in 40 cases (72.2%) and more than 90% in only 8 cases (14.5%) while no comment was mentioned in the pathology report for the rest 7 cases of the study, the overall survival time in correlation with tumour necrosis of the primary tumour for patient > 90% compared with those < 90% was 88.7 and 52.2 months respectively with statistically significant *p*-value (0.017).This result was similar to the study done by [13] where histological response of the primary tumour to neoadjuvant chemotherapy was a well-recognized prognostic factor in patients with metastatic osteosarcoma. In another study where 77 pediatric patients diagnosed with metastatic osteosarcoma, they underwent pulmonary metastasectomy, chemonecrosis had a significant higher 5-year survival rate in patients > 90% when compared with those with chemonecrosis < 90% (*p*-value 0.008) [14]. The Livestrong Young Adult Alliance has conducted a meta-analysis of individual patient data from prospective neoadjuvant chemotherapy osteosarcoma studies and registries, the study was published by American Society of Clinical Oncology in which 4838 patients were included with median age 15 years old, data were collected from 5 international cooperative groups, the results revealed significant relation between survival and response to chemotherapy *p* = 0.001 [15].

On the other hand, an Irish study done by O'Kane et al. reviewed 97patients diagnosed with localized and metastatic osteosarcoma (lungs and other sites) with a median age 23 years old, the 33 patients who achieved ≥ 90% primary tumour necrosis, the 5-year OS was 82% while the 29 patients who had < 90% tumour necrosis, the 5-year OS was 68% with statistically insignificant impact of tumour necrosis on survival *p* = 0.15 [16].

Regarding pulmonary metastases, size of metastatic nodules in this study ranged from (0.5 to 10 cm) with a mean 3.4 cm, the 5-year overall survival in patients with pulmonary nodules 2 cm or less was 83.1% compared to 69.7% in patients with pulmonary nodules more than 2 cm with statistically insignificant *p*-value (0.22).This is similar to the study which was carried out in Japan, where data was gathered from 37 patients with pulmonary metastasis from osteosarcoma who underwent metastasectomy. They found no statistical significance between maximal diameter of the lung nodules and overall survival [17].

Regarding the number of pulmonary nodules, the number in our study was ranging from (1to 28) with a median of 4. The 5- year overall survival in patients with three pulmonary nodules or less was 77.2% compared to 71.8% in patients with pulmonary nodules more than three with statistical insignificance (*p* = 0.313). The International Registry of Lung Metastases retrospectively reviewed 575 patients who underwent 708 lung metastasectomies. They confirmed that completeness of surgery resection, histology, and DFI as independent prognostic factors while number of metastases, presence of lymph node metastases, surgical approach, and number of metastasectomies did not statistically influence long-term survival [18].

In the current study patients, 76.4% had bilateral metastatic nodules with 5-year OS 69.4% and 23.6% had unilateral nodules with 5-year OS 92.3% with statistical insignificant difference between overall survival and laterality (*p* = 0.076). In our patients, 76.4% had bilateral metastatic nodules with 5-year OS 69.4% and 23.6% had unilateral nodules with 5-year OS 92.3% with statistical insignificant difference between overall survival and laterality (*p* = 0.076). That was similar to studies done by (Harting and Blakely, 2006; Chen et al., 2009). In a more recent study done by Okiror et al. (2016) during the period between August 2007 and January 2014, a total of 80 pulmonary metastasectomies were performed on 66 patients with metastatic sarcoma, there were no postoperative in-hospital deaths, the median age was 51 years (range, 16–79), fourteen patients had bilateral lung operations and surgical access was by video-assisted thoracoscopic surgery in 48 (73%) cases, the median number of metastases resected was 3 (range, 1–9), there was no significant difference in survival between patients with high-grade versus low-grade tumors (*p* = 0.13), histological type (osteosarcoma vs. other soft tissue sarcoma types, *p* = 0.14), unilateral versus bilateral lung metastases (*p* = 0.48). On the other hand, Other investigators demonstrated that patients with bilateral lung metastases had reduced overall survival when compared to patients with unilateral disease [19, 20, 21], also the study done by Tronc et al*. *[3] in which 52 pediatric patients underwent PM, they concluded that there was a statistically significant difference in survival rates between patients with unilateral metastases and those with bilateral metastases (49% vs 7%, *p* = 0.001) [22–24].

About surgical margin of metastatic nodules in our study, 32.7% of patients were excised with negative margins, 34.5% were excised with positive margins and 32.7%were excised with close margins, the 5-year OS was 94% in negative margins, 62% in positive margins and 87% in close margins. In our study, there was a tendency towards longer survival in patients with negative margins compared to patients with close and positive margins but the difference between OS and surgical margin was not statistically significant (*p *= 0.08), This may be attributed to small number of the studied patients. Similar conclusion was reached by Tanju et al. study, which was carried out in Turkey, where they analysed the role of extended resections if it may be necessary to achieve tumour-free borders for secondary pulmonary malignancies, and they found no statistical significance [25]. In another Jordanian study, King Hussein Cancer Center, the patients with positive resection margins in any of the resected nodules did not have statistically significant differences in OS compared to patients with negative resection margins [26]. On the other hand, Kim et al. at Massachusetts General Hospital, Harvard Medical School, USA, they studied 97 patients who underwent pulmonary resection for metastatic sarcoma, they proved that tumour resectability for pulmonary metastasis for sarcoma can be associated with prolonged survival *p* value (0.004) [27].

According to the time of diagnosis of pulmonary metastasis of our participants: 23.6% of patients developed synchronous metastasis with 5 year OS 70%, 29.1% of patients developed metastasis during treatment with 5 year OS 52.9%, 32.7% of patients developed metastasis within the first year follow up after end of treatment with 5-year OS 67% and 14.5% presented with metastatic after the first year follow up with 5-year OS 100%. There was statistically significant difference between OS and metastatic free period in our study (*p* = 0.001). This agrees with the study which included seventy-seven pediatric patients with metastatic osteosarcoma were analysed, they reported that regarding timing of lung metastasis, both presence of lung metastases at diagnosis or during follow-up were found to correlate with overall survival *P *= 0.004 and *P* = 0.003 respectively [12]. In another study, they observed a significant association between the timing of detection of metastasis in relation to chemotherapy and survival *P* < 0.0001 [13].

Surgical approaches of pulmonary metastasectomy among the studied patients had the following distribution: 90.9% of patients did metastasectomy via thoracotomy, 3.6% of patients via VAT and 5.5% did both. Open thoracotomy is the most common surgical approach, this was reported [10].

Regarding the type of metastasectomy in the present study, (81.8%) of patients underwent metastatectomy (wedge resection), lobectomy was done in (14.5%) of patients and Only (3.6%) of patients underwent pneumonectomy, the 5 year OS was 77% in patients who did wedge resection and it was 64.8% in patients who did lobectomy and pneumonectomy with statistically insignificant *p*-value = 0.6. This is in agreement with the study done by MD Anderson Cancer Center published in Journal of Pediatric Surgery, which included 115 pediatric patients with pulmonary metastasis secondary to osteosarcoma, revealed that there was no significant difference in 3 year overall survival when comparing lobectomy to wedge resection (18% vs 30%) *p* = 0.91 [14].

In our study, some patients underwent repeated thoracotomies either sequential thoracotomy for bilateral lung metastases or for recurrent lung metastasis, the mean number of thoracotomies was 2, the number was ranging from 1 to 6 thoracotomies, there was no statistically significant correlation between survival and the number of thoracotomies *p* value 0. 097. This result was similar to the study done [28].

In our study, complications related to pulmonary metastasectomy were presented in 19 cases in the form of (lung collapse, pleural effusion, pneumothorax and surgical emphysema), there were no perioperative deaths.

The overall median survival time in our study was 64 months duration and the 5-year overall survival was (74.8%). The 5-year overall survival in our Osteosarcoma patients was 68%. By reviewing other studies we found a retrospective study included 68 patients of children and adults who underwent curative pulmonary resection for metastatic lung tumour from different solid tumours, it was published in European Journal of Cardio-thoracic surgery, the overall 5 year survival rate after pulmonary metastasectomy was 75.7% [29] which is similar to our results, Another study included 210 children and young adults with a diagnosis of metastatic bone and soft tissue sarcoma the 3-year estimates of OS of all 210 patients included in the study was 74.94.1% [30]. A recent European study which was published in Journal of Thoracic Diseases under the name of Metastasectomy in pediatric patients, the study mentioned that ranges of overall survival vary from 20 to 70% [31].

## Conclusion

We concluded that the resection of pulmonary metastases of paediatric solid tumours is a safe and potentially curative treatment. Good surgical candidates for pulmonary resection are those showing a long metastatic free period and unilateral pulmonary metastases. Five-year survival is influenced by resectability, primary tumour necrosis and timing of appearance of pulmonary metastasis. Repeat resection for recurrent lung metastases is recommended.

## Recommendations

Multidisciplinary treatment involving pediatric oncologists, surgeons, and radiation oncologists is necessary to obtain positive results in children who have pulmonary metastases and for better selection of those who will benefit from pulmonary metastatectomy.

Computed tomography (CT) scan is considered the gold standard for the identification of pulmonary nodules but it is also important to add more effective examinations as positron emission tomography (PET) which had a sensitivity and accuracy in detection of occult distant metastases or recurrence at the primary site to avoid unnecessary lung resections. We should perform re-evaluation of metastases using these devices before pulmonary surgery.

## Data Availability

The datasets used/analyzed during this study are available from the corresponding author on request.

## Abbreviations

AFP: Alpha-fetoprotein
ALP: Alkaline phosphatase
AMD: Actinomycin
CBC: Complete blood picture
CEA: Carcinoembryonic antigen
COG: Children’s Oncology Group
COSS: Cooperative Osteosarcoma Study Group
CRCs: Colo-rectal cancers
CT: Computed tomography
DFI: Disease-free interval
EFS: Event free survival
EOI: European Osteosarcoma Intergroup
ESTS: European Society of Thoracic Surgery
H&E: Haematoxylin and eosin
IRLM: International Registry of Lung Metastases
KFT: Kidney functions tests
LDH: Lactate dehydrogenase
LFT: Liver functions tests
MAP: Methotrexate /adriamycin /platinol
MR: Minor Response
MTX: Methotrxate
NCI: National Cancer Institute
NRSTS: Non-rhabdo soft tissue sarcoma
OS: Osteosarcoma
OS: Overall survival
PD: Progressive Disease
PM: Pulmonary metastasectomy
PR: Partial Response
PREFS: Post relapse event free survival
PRS: Post-Relapse Survival
R: Resection margin
RMS: Rhabdomyosarcoma
SALP: Serum alkaline phosphatase
SD: Stable Disease
SEER: Survival Epidemiology and End Results
SIOP: International Society of Pediatric Oncology
SIOPEL: Society of Pediatric Oncology Liver Tumor Study Group
SJCRH: St. Jude children’s research hospital
SS: Synovial sarcoma
STSs: Soft tissue sarcomas
TN: Tumor necrosis
VATS: Video-assisted thoracoscopic surgery
VEGFR1: Vascular endothelial growth factor receptor 1
XRT: Radiotherapy
